# A novel protocol to induce mental fatigue

**DOI:** 10.3758/s13428-023-02191-5

**Published:** 2023-08-03

**Authors:** E. K. Hassan, A. M. Jones, G. Buckingham

**Affiliations:** Public Health and Sport Sciences, University of Exeter, St Luke’s Campus, Heavitree Road, Exeter, EX1 2LT UK

**Keywords:** AX-CPT, BRUMS, Cognitive fatigue, Methodology

## Abstract

Mental fatigue is a commonplace human experience which is the focus of a growing body of research. Whilst researchers in numerous disciplines have attempted to uncover the origins, nature, and effects of mental fatigue, the literature is marked by many contradictory findings. We identified two major methodological problems for mental fatigue research. First, researchers rarely use objective measures of mental fatigue. Instead, they rely heavily on subjective reports as evidence that mental fatigue has been induced in participants. We aimed to develop a task which led to not only a subjective increase in mental fatigue, but a corresponding performance decrement in the mentally fatiguing task as an objective measure. Secondly, current mental fatigue paradigms have low ecological validity – in most prior studies participants have been fatigued with a single repetitive task such as the n-back or Stroop. To move towards a more ecologically valid paradigm, our participants undertook a battery of diverse cognitive tasks designed to challenge different aspects of executive function. The AX-CPT, n-back, mental rotation, and visual search tasks were chosen to challenge response inhibition, working memory, spatial reasoning, and attention. We report results from 45 participants aged 19 to 63 years who completed a two-hour battery comprising four different cognitive tasks. Subjective fatigue ratings and task performance were measured at the beginning and end of the battery. Our novel method resulted in an increase in subjective ratings of fatigue (*p* < 0.001) and a reduction in task performance (*p* = 0.008). Future research into mental fatigue may benefit from using this task battery.

## Introduction

The concept of mental fatigue is one which is intuitively understood from a lay perspective. For many of us, mental fatigue may be familiar as a sensation we experience occasionally at the end of a particularly challenging day. For others who work in jobs which are routinely demanding, such as surgeons, those in the military, or air traffic controllers, mental fatigue may be much more commonplace. It is important that we understand how mental fatigue may affect our lives. However, researchers across multiple disciplines do not yet agree on a scientific definition of mental fatigue and, crucially, how we should induce it experimentally.

Research into mental fatigue and allied concepts (e.g., cognitive fatigue, ego depletion, self-control exertion) has taken numerous different conceptual approaches (Pattyn et al., 2018) leading to differences in understanding between researchers in different disciplines (Baumeister, 2020; Forestier & Chalabaev, 2020). For example, mental fatigue has been conceptualized both as having a variety of effects (Lorist & Faber, 2011), and as being highly task-specific (e.g. Tanaka et al., 2012). Some researchers consider ego depletion as a type of mental fatigue (e.g. Habay et al., 2023), whereas others argue that they are separate phenomena (e.g. Forestier & Chalabaev, 2020). There are similar differences in opinion about the relationship between boredom and mental fatigue (e.g. Pattyn et al., 2008; Smith et al., 2019). Pattyn et al. (2018) describe these differing accounts of mental fatigue as “mainly semantic”. By operationalizing mental fatigue using different definitions, researchers limit the value of their research and put researchers in other disciplines at risk of “reinventing the wheel” (Skau et al., 2021).

Consequently, the methods used to induce mental fatigue are highly varied (Pitts & Bhatt, 2023; Sun et al., 2021; Tran et al., 2020; Van Cutsem et al., 2017). Some researchers use simple tasks such as the Stroop color–word test, where participants must exercise inhibition to overcome semantic interference (Pageaux et al., 2015; Smith et al., 2016; Thompson et al., 2020) or variations of an n-back task, which require participants to remember differing lengths of sequences of items (numbers, letters, or images) whilst monitoring incoming stimuli (Clark et al., 2019; Shortz et al., 2015). Other more complex tasks have also been used to try to induce mental fatigue. For example, O’Keeffe et al. (2020) compared a ‘TloadDback’ task, designed to maintain alertness whilst challenging each participant at an individualized level of difficulty, with the A-X Continuous Performance Test (AX-CPT), designed to test memory. O’Keeffe et al. (2020) demonstrated that the nature of the mental fatigue that is induced by these two tasks differs. They found that participants’ physiological arousal was affected by task choice, with participants showing significantly greater galvanic skin response and lower heart rate variability during the TloadDback than during the AX-CPT. Subjective effects also differed, with participants reporting significantly lower sleepiness, higher end-point motivation, and significantly higher vigor after the TloadDback compared to after the AX-CPT. Mental fatigue responses also differed, with participants reporting higher mental fatigue after the TloadDBack when measured by a visual analogue scale, but higher mental fatigue after the AX-CPT when measured by the Brunel Mood Scale (Terry et al., 2003). These results highlight the importance of task choice when inducing mental fatigue, and it is reasonable to assume that this issue extends beyond the tasks tested in their study.

Differences in the tasks used by researchers to induce mental fatigue may partly explain the varying results in the literature (Holgado et al., 2020). The issue of task specificity is already the subject of fierce debate in the allied ego depletion literature, where failed replications and reduced effect sizes have been attributed to the failure by researchers to select tasks which generate the motivational conflict required to induce ego depletion (Baumeister, 2019, 2020; Forestier et al., 2022; Forestier & Chalabaev, 2020). The duration of mental fatigue-inducing tasks also varies greatly – from four minutes (Bray et al., 2011) to 100 minutes (Budini et al., 2014) – which could affect the outcome of the manipulation (MacMahon et al., 2021). Research into this issue has so far been inconclusive, with some researchers proposing shorter tasks as more appropriate for inducing mental fatigue (O’Keeffe et al., 2020), and others deeming it necessary to use longer tasks, with Van Cutsem et al. (2017) notably excluding short-duration tasks from their meta-analysis. Research focusing on the differing influences of tasks of different durations is ongoing (Dallaway et al., 2022). Whilst the implications of using different task durations are not as easily apparent as the implications of using different tasks, introducing methodological heterogeneity in this way is only likely to make it more difficult to elucidate the nature of mental fatigue (Arber et al., 2017).

Despite the various conceptual and methodological approaches in the literature examining the origins, characteristics, and effects of mental fatigue, two main limitations are apparent. The first limitation is an over-reliance on subjective measures of fatigue at the expense of other measures. By adopting the definition of mental fatigue as a “psychobiological state caused by prolonged periods of demanding cognitive activity and characterized by subjective feelings of ‘tiredness’ and ‘lack of energy’” (Marcora et al., 2009), we can determine whether participants are experiencing mental fatigue by subjecting them to a demanding cognitive activity and taking subjective measures. Subjective measures alone are, however, problematic due to the potential for participants to respond to experimenter demands. Additionally, tiredness and a lack of energy are not unique markers for a sense of mental fatigue and could be conflated with boredom or other sensations (Pattyn et al., 2008), including physical fatigue. Rather than relying on subjective measures alone, for participants to be mentally fatigued there should be concurrent evidence that a high cognitive demand has been placed on participants. One way of determining whether a cognitive activity is demanding is to examine performance in the cognitive activity. If participants’ performance declines over time, then this shows that the task is demanding enough that participants fail to maintain task performance as time progresses. It is crucial to use both types of measures concurrently, as examining one measure at the exclusion of another risks oversimplifying mental fatigue (Smith et al., 2019) as well as risking error if bias can affect the measure (for alternative perspectives on these criteria, see Pageaux et al. (2014) and Van Cutsem et al. (2017)). In line with this perspective, researchers have employed various subjective, physiological, and behavioural measures in an effort to better understand how mental fatigue manifests (Dallaway et al., 2022; Tanaka et al., 2014).

The second major limitation in the current literature is that the paradigms which are most frequently used have low ecological validity (Gantois et al., 2021). Tasks such as the Stroop, n-back, and AX-CPT were specifically designed to target a limited number of cognitive processes in order to understand those processes better. For example, the Stroop task was designed specifically to test semantic interference (Stroop, 1935), which would not typically be experienced with great repetition or at great length as a part of daily life. When we think about the subjective experience of a few hours of challenging work, it is qualitatively different to the subjective experience of doing a single specific and repetitive cognitive test for 30, 60, or 90 min. Rather, doing multiple different types of tasks and having to switch between them is more reflective of the demands of a typical day. Task-switching between multiple short-duration tasks also places additional demands on participants, increasing the likelihood that they will experience mental fatigue (Dang et al., 2013).

Without addressing these two weaknesses and moving towards a consistent way of inducing mental fatigue, researchers in all disciplines who are interested in elucidating the nature, origins, and effects of mental fatigue are likely to produce and build upon heterogenous outcomes (Holgado et al., 2020), which cannot be interpreted in a way that is meaningful for real-world applications. This will hinder our understanding of mental fatigue and its effects, which is problematic given the importance for certain populations, such as athletes, night-shift workers, emergency services, pilots, and those working in other demanding environments e.g., the armed forces.

The aim of this study is to develop a novel and more ecologically valid method for inducing mental fatigue which causes performance decrements as well as a subjective increase in fatigue. To this end, we developed a task battery based on a number of cognitive tests. Our hypotheses are that completing two hours of this cognitive task battery will cause both (a) an increase in subjective feelings of fatigue, and (b) a decrement in cognitive task performance.

## Methods

### Materials

#### Cognitive tasks

Two custom programs were developed in PsychoPy3 (v2020.2.4, Peirce et al., 2019) and designed to run full-screen in browser in Pavlovia (https://pavlovia.org/; accessed February 2021). One program was a training program designed to familiarize participants with the experimental procedures. The other program was a testing program designed to elicit mental fatigue. Both programs were designed to be used on a laptop or desktop computer with participants responding using a keyboard, with the full-screen design intended to hide distracting information such as the time or computer notifications. The files containing code and materials for each program can be found at https://osf.io/357un/ and are free to use under a General Public License v3.0 (https://psychopy.org/about/index.html; https://github.com/psychopy/psychopy/blob/release/LICENSE).

Both programs consisted of four different tasks: the AX-CPT, an n-back task, a visual search task, and a mental rotation task. The tasks were selected based on their prior use in the mental fatigue literature, and in order to maximise the breadth of executive functions which would be challenged: the AX-CPT task requires participants to engage their short-term (working) memory and inhibit their responses (Barch et al., 1997); the n-back task requires participants to continuously update the information stored in working memory and sustain attention (Kirchner, 1958); the visual search task requires participants to control spatial attention (Horowitz & Wolfe, 1998); and the mental rotation task requires spatial reasoning (Ganis & Kievit, 2015). Whilst the Stroop task is commonly used in the mental fatigue literature as previously outlined, we chose not to include it as the primary functions which it challenges – response inhibition, semantic interference, and attentional control – are all challenged in other tasks selected. The Stroop task also requires different types of responses to the other tasks selected which would have added additional complexity to the task with unknown effects on the Stroop task itself and any task that immediately followed it.

##### AX-CPT

**Fig. 1:**
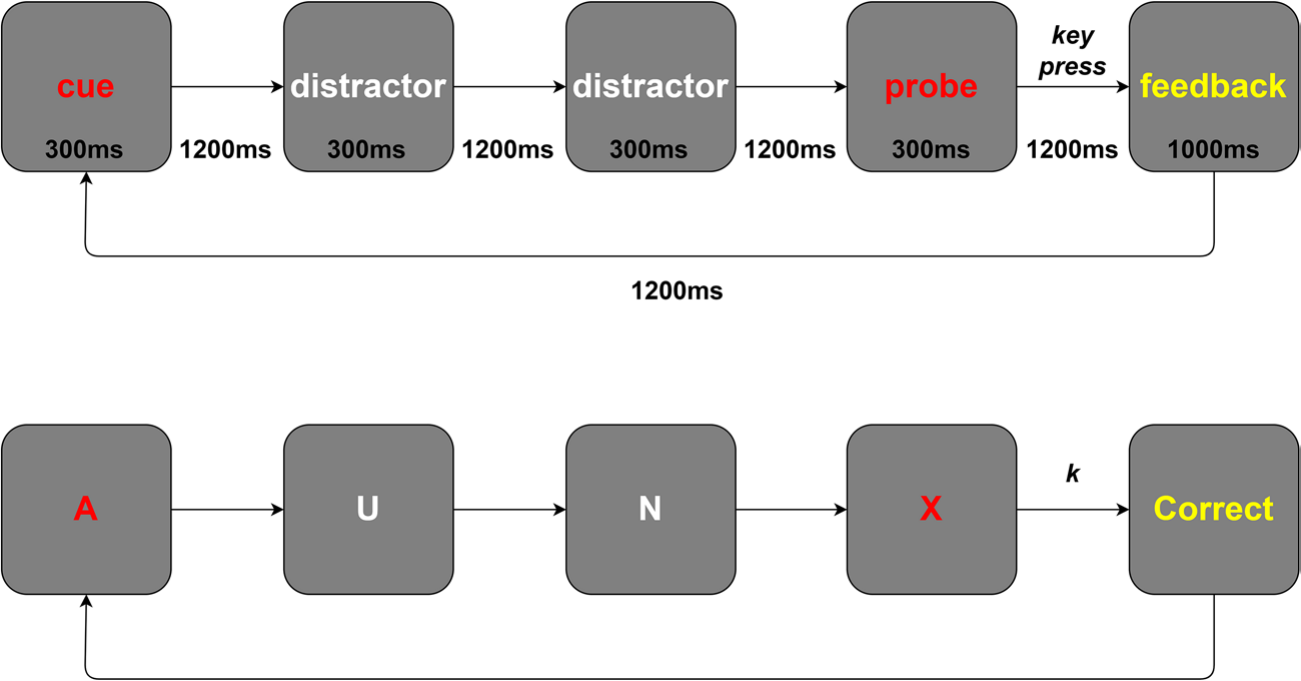
AX-CPT procedures and example target trial with correct response (‘k’)

The AX-CPT has been used extensively to induce mental fatigue and was designed in line with Marcora et al. (2009). In each trial, participants saw a series of four letters consisting of a cue, two distractors, and a probe. The cue was shown in red and could be any letter other than K or Y. Distractors were shown in white and could be any letter other than A, K, X, or Y. The probe was shown in red and could be any letter other than K or Y. There were four different types of trial. In target trials, A was the cue and X was the probe. The three non-target trials followed a B-X, A-Y, or B-Y cue-probe sequence, where B and Y represent any possible letter other than A or X. As in Marcora et al. (2009), 70% of trials were target trials and 30% were non-target trials (10% of each type). The correct response was ‘k’ in target trials and ‘d’ in non-target trials. Target and non-target trials were presented in a pseudorandom order, where in each ten trials, seven were target trials and there was one each of each type of non-target trial. Each letter was shown centrally on a grey background and was normalized to 7.5% of the height of the participant’s screen. The letters were each shown for 300 ms with a 1200 ms interval immediately afterwards, during which a blank screen was shown. After the probe had been presented and either participants had responded or 1200 ms had elapsed, feedback was given in yellow text. If participants responded correctly, “Correct” was shown for 1000 ms. If participants failed to respond in time or responded incorrectly, “Incorrect” was shown for 1000 ms. After a 1200 ms blank screen, a new cue was shown (Fig. 1).

##### N-back

**Fig. 2:**
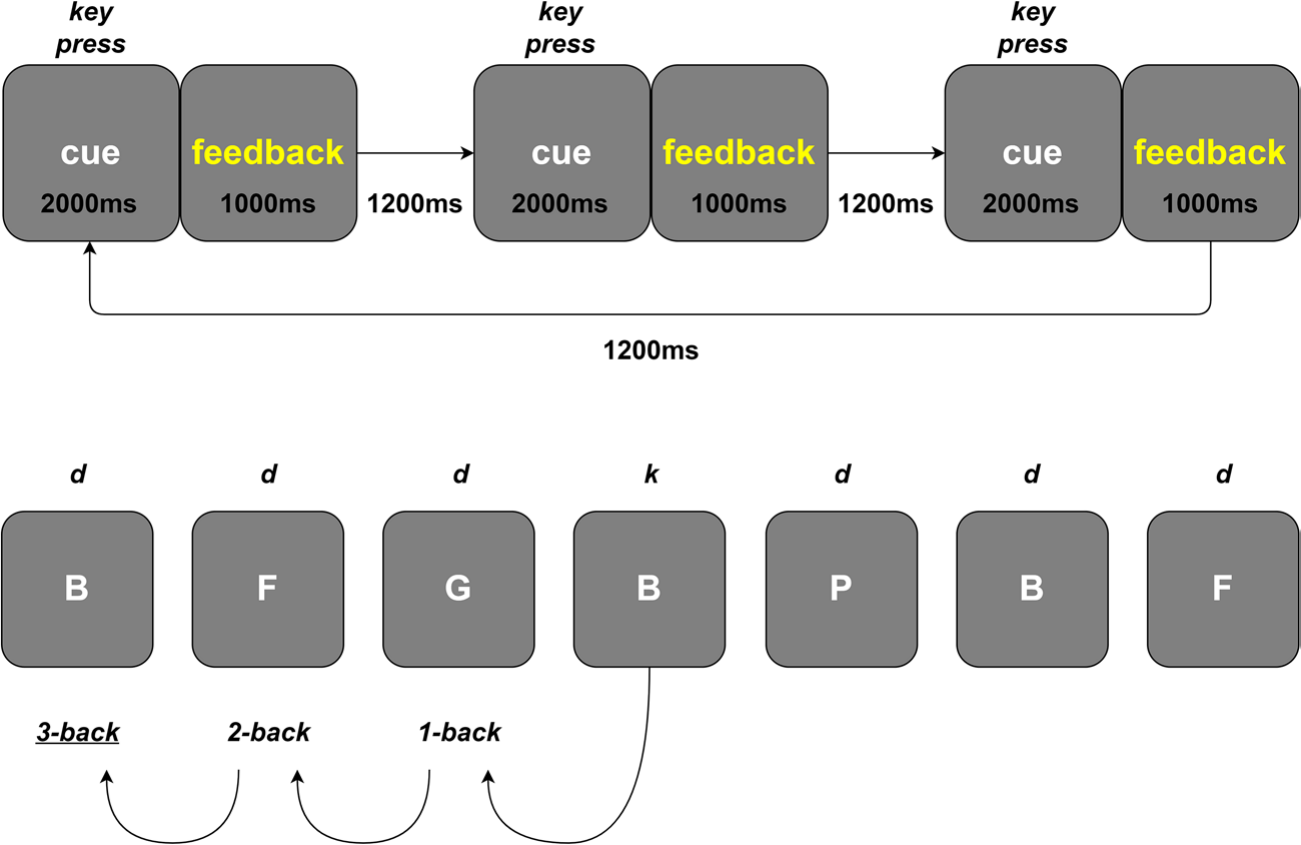
N-Back procedures and example series of letters with correct responses indicated above

In the n-back task, participants are shown a series of items and are required to indicate whether the current item is the same as the item shown *n* items previously (Kirchner, 1958). We used a 3-back task where participants had to indicate whether the current letter was the same as the letter shown 3 letters ago. Participants responded ‘k’ if it was the same (30% of trials) and ‘d’ if it was not (70% of trials). Trials were presented in a pseudorandom order, where in each 10 trials, three were target trials and seven were non-target trials. Each letter was presented for 2000 ms, followed by 1000 ms of feedback and a 1200 ms interval as in the AX-CPT task (Fig. 2). All of the letters were shown in a white font on a grey background at the same height as the letters in the AX-CPT task. Whilst prior research has often used the 2-back version of the n-back, we wanted to maximise the cognitive demands of the task battery in order to induce fatigue, and so chose the more demanding 3-back. Additionally, pilot research (available at https://osf.io/zgchx) indicated that participants did not find the 2-back challenging when performed continuously for 30 minutes.

##### Visual search task

**Fig. 3:**
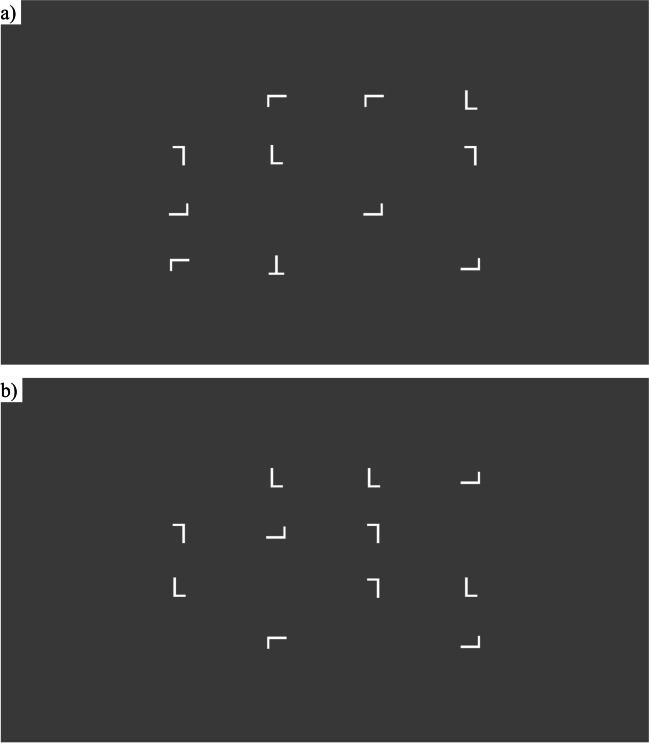
Examples of the **a** target and **b** non-target visual search trials, with **a** showing T in the bottom row and rotated by 180 degrees

We used a visual search task based on that used by Horowitz & Wolfe (Horowitz & Wolfe, 1998). In this task, participants were shown 11 letters on a screen which could be rotated either 0, 90, 180, or 270 degrees. The letters could appear in any space on an invisible 4 x 4 grid. The grid was cantered and the size was normalized so that it would leave a border of 25% of the participants’ screen size on all sides. Every trial consisted of at least ten letter Ls. Half of the trials were target trials where a letter T was also present in the grid in a random orientation and position (Fig. 3a). The other half of the trials were non-target trials where an additional letter L was present (Fig. 3b). Trial order was pseudorandomized so that in every ten trials, five were target trials and five were non-target trials. Participants were told to press ‘k’ when the T was shown and ‘d’ otherwise. There was no time limit for participants to respond. Once they had responded, 1000 ms feedback and a 1200 ms interval were shown as in the AX-CPT and n-back tasks. Letters were presented as in the n-back task.

##### Mental rotation task

In mental rotation tasks, participants are presented with an image of two items and asked to determine whether the items match. One item may be rotated in relation to the other, requiring participants to visualize and “mentally rotate” one or other of the items to complete the task (Shepard & Metzler, 1971).

**Fig. 4:**
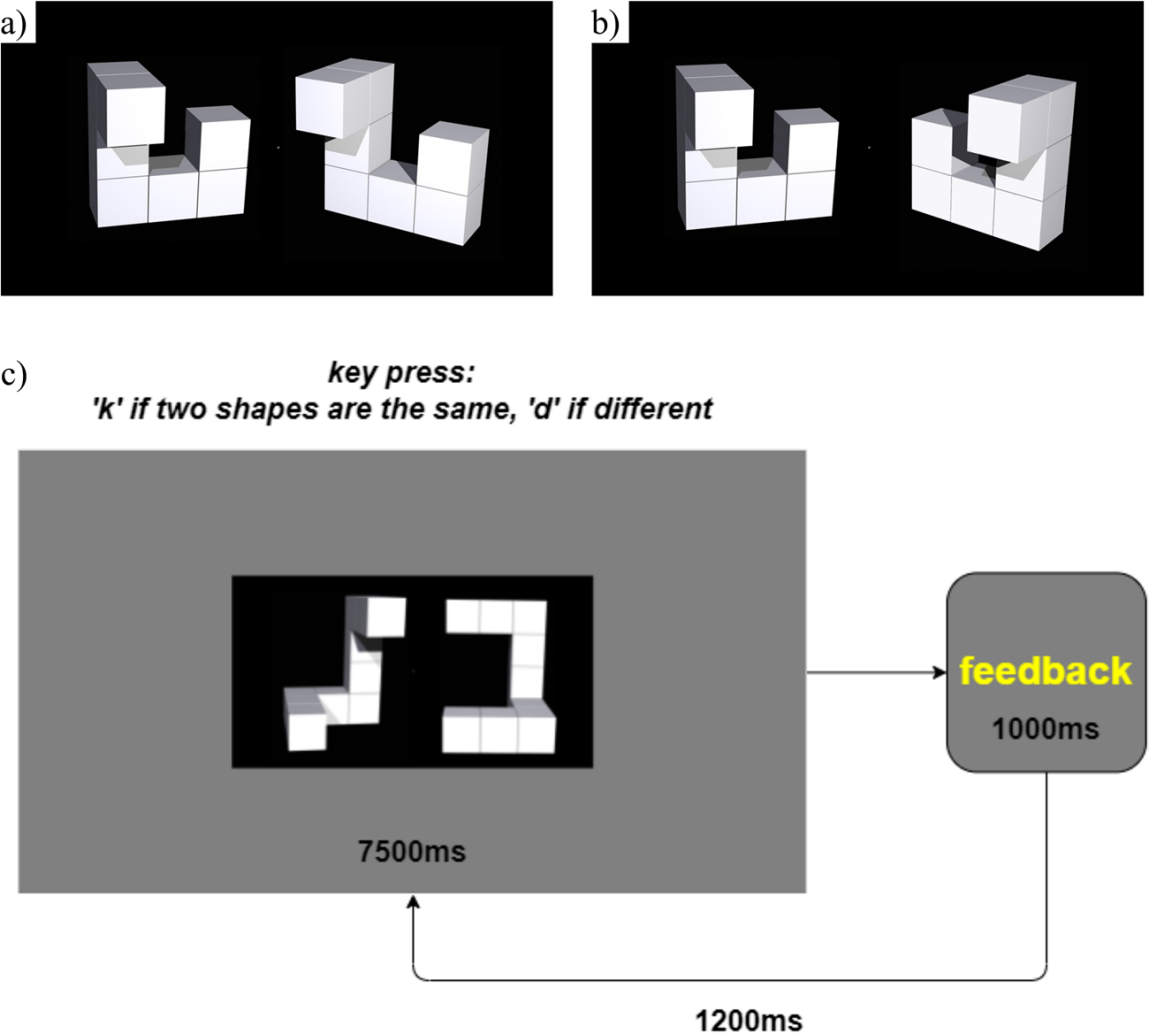
Example stimuli and procedures in the mental rotation task. **a** Example matching stimulus, with shape on the right rotated by 50 degrees on the vertical axis relative to the shape on the left (stimulus ‘1_50’ from Ganis & Kievit, 2015). **b** Example non-matching stimulus, with pseudo-mirror shape on right rotated by 50 degrees on the vertical axis relative to the shape on the left (stimulus ‘1_50_R’ from Ganis & Kievit, 2015). **c** Procedures: participants were presented with two shapes for up to 7500 ms. They were instructed to respond by pressing ‘k’ if the two shapes were the same and ‘d’ if they were different. This was followed by 1000 ms of feedback and a 1200 ms interval

We used a set of stimuli developed and validated by Ganis & Kievit (2015). In Ganis & Kievit’s (2015) stimuli, the left-hand shape is always oriented in the same way, with the right differing by 0, 50, 100, or 150 degrees around a vertical axis (25% of the set differs by each amount). Half of the stimuli show matching shapes (Fig. 4a) and half show non-matching shapes (Fig. 4b). The non-matching shapes are pseudo-mirror images which are made of the same number of cubes and have the same configuration of arms as the matching shapes. We chose to use the same subset of 96 stimuli as Ganis & Kievit (2015) used in their validation study. With the subset of 96 stimuli, a mean response time of ~ 3000 ms (the largest mean response time in Ganis & Kievit, 2015) would mean that each trial would take 5.2 s allowing for each participant to complete at minimum 115 trials. As the stimuli were presented in a pseudorandom order requiring that every stimulus had been presented at least once before any could be presented again, a participant would see each of the 96 stimuli at least once in 115 trials. Stimuli were presented centrally at 50% of the width and 25% of the height of the participant’s screen. Participants were presented with each stimulus for 7500 ms and required to respond with ‘k’ if the shapes matched and ‘d’ if they did not. As in the other tasks, participants received written feedback for 1000 ms, followed by a 1200 ms interval (Fig. 4c).

#### Brunel Mood Scale (BRUMS) Questionnaire

To assess the subjective experience of fatigue, a computerized version of the Brunel Mood Scale (BRUMS; Terry et al., 1999, 2003) was developed. The BRUMS comprises 24 items with descriptors such as “bitter”, “active”, and “uncertain” which can be equally divided onto six subscales – anger, confusion, depression, fatigue, tension, and vigor. Participants reported how they were feeling at that moment by rating each item from 0 (Not at all) to 4 (Extremely) using a mouse to select one of five points distributed equally along a horizontal line. The total score for each subscale was calculated by adding up the numbers selected for each item, giving a possible range for each subscale from 0 to 16.

### Participants

We recruited 71 participants over the age of 18 who self-reported being free from cognitive and/or visual impairments. The sample size was determined by feasibility constraints – namely the financial cost of renumerating participants, and the availability of researchers to run the study. Data collection ceased when the budget for the study was nearly depleted and when there were no researchers available to continue running the study. Informed consent was obtained from all study participants. This study was approved by the University of Exeter Sport and Health Sciences Ethics Committee (Reference 201021-A-07) and was conducted in line with the Declaration of Helsinki. Participants were paid £20 to compensate them for their time taking part in the study.

Of the 71 participants recruited, 12 withdrew (five failed to complete the initial training session and seven failed to complete the testing session). Five of these participants withdrew due to technical errors during data collection which led to a failure to complete the task or save the data.

Nine participants were excluded due to poor data quality. We decided the parameters for what constituted poor data quality prior to data collection to reduce the possible effects of decision-making bias on the outcomes of the study, but we did not preregister these parameters. We excluded participants if they failed to give any response on more than 25% of trials in the cognitive tasks, or responded with only ‘k’ or ‘d’. We also excluded participants if they rated every item in the BRUMS 0 or 4, or if they completed the BRUMS in less than one second per item. Of the nine participants who were removed, none were excluded based on their responses in the BRUMS. A summary of the responses of the excluded participants can be seen at https://osf.io/6arv9/.

Following these withdrawals and exclusions, our final sample consisted of 45 participants aged 19 to 63, with an average age of 35.47 ± 13.59.

### Training protocol

Once participants had given informed consent, they were sent a link to the training program. In the training program, participants were given full instructions and the names of each cognitive task and practiced each task for five min with self-paced breaks between tasks. Participants practiced the AX-CPT first, followed by the n-back task, the visual search task, and the mental rotation task.

### Mental fatigue protocol

Once participants had finished the training and this had been verified by the experimenter, they were sent a link to the testing program. Participants were instructed to do the testing program as close to 48 h following the training session as possible and to ensure that they would be in a quiet environment where they would not be interrupted. They were also advised that they may be mentally fatigued following the testing session and that they should allow time for rest afterwards.

**Fig. 5:**
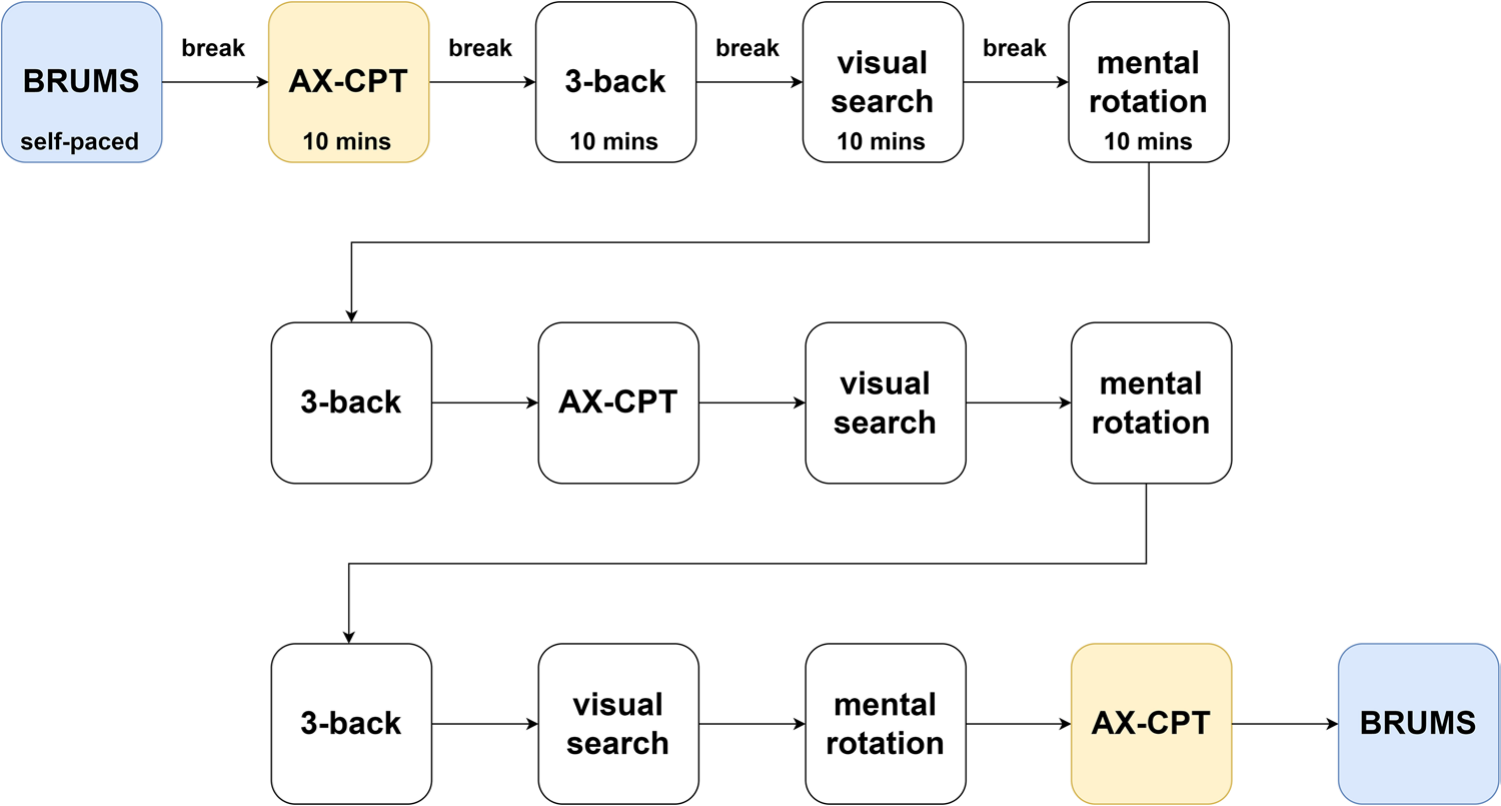
The mental fatigue protocol. Participants completed a BRUMS questionnaire followed by three repeats of four different cognitive tasks: the AX-CPT, a 3-back task, a visual search task, and a mental rotation task. Each cognitive task was completed for 10 min at a time with optional self-paced breaks between tasks. After all the cognitive tasks had been completed, the BRUMS was administered again. The *blue* and *yellow* boxes highlight stages where data was collected to test hypotheses (a) and (b), respectively

At the beginning of the testing program, participants completed a digitized version of the BRUMS questionnaire. Participants then completed each task three times for 10 min for a total of 120 min time on task (Fig. 5). The AX-CPT was chosen as the “critical task” in which to measure task performance, as in Marcora et al. (2009). Consequently, the AX-CPT was first and last. The remaining tasks were presented in a pseudorandom order. The order of the tasks was chosen so that participants did not repeat tasks back-to-back; to maximize the time between tasks being repeated so that the procedure felt less repetitive; so that the time between tasks being repeated was similar between the different tasks; and so that if any order effect was present it would be the same for all participants. A self-paced break was placed between each task. In this break, participants were shown brief instructions telling them which task would begin next and reminding them how to complete the task. They were also reminded to respond as quickly and accurately as possible.

### Statistical analysis

We conducted data cleaning, visualization, and analysis in RStudio (Rstudio 2022.02.0+443 “Prairie Trillium” Release) using here (Müller & Bryan, 2020), dplyr (Wickham et al., 2017), stats (R Core Team, 2022), data.table (Dowle & Srinivasan, 2019), psych (Revelle, 2018), gtools (Warnes et al., 2018), ggplot2 (Wickham, 2016, p. 2), car (Fox & Weisberg, 2011), tidyr (Wickham & Henry, 2018), gridExtra (Auguie & Antonov, 2017), and grid (R Core Team, 2022). All scripts and data, including data formatted for use with other statistical software, are available at https://osf.io/6hjc3/.

For our primary hypotheses, we used two Wilcoxon signed-rank tests to analyse the data. Both tests had time point (pre/post) as the independent variable. To assess subjective feelings of fatigue, BRUMS fatigue subscale score at the beginning and end of the testing program (highlighted in blue, Fig. 5) was used as the dependent variable. To assess task performance in the AX-CPT, we chose to integrate reaction time and accuracy in order to control for participants changing their strategy as they become fatigued and prioritizing one or the other of the two performance metrics (van der Linden, 2011). To do this, we used the balanced integration score (BIS; Liesefeld & Janczyk, 2019). The BIS combines reaction time and accuracy into a single metric which is standardized across all conditions (in this case, time points) and participants. A BIS score of zero represents an average level of performance across all participants and conditions, with above average and below average performance indicated by positive and negative numbers, respectively. The BIS was chosen as it has been shown to be the least sensitive to speed–accuracy trade-offs in comparison to other integrated measures of reaction time and accuracy (Liesefeld & Janczyk, 2019). The BIS score was calculated using the reaction time and response data collected during the first and last repeat of the AX-CPT task (highlighted in yellow, Fig. 5).

## Results

### BRUMS Fatigue Subscale Ratings

**Fig. 6:**
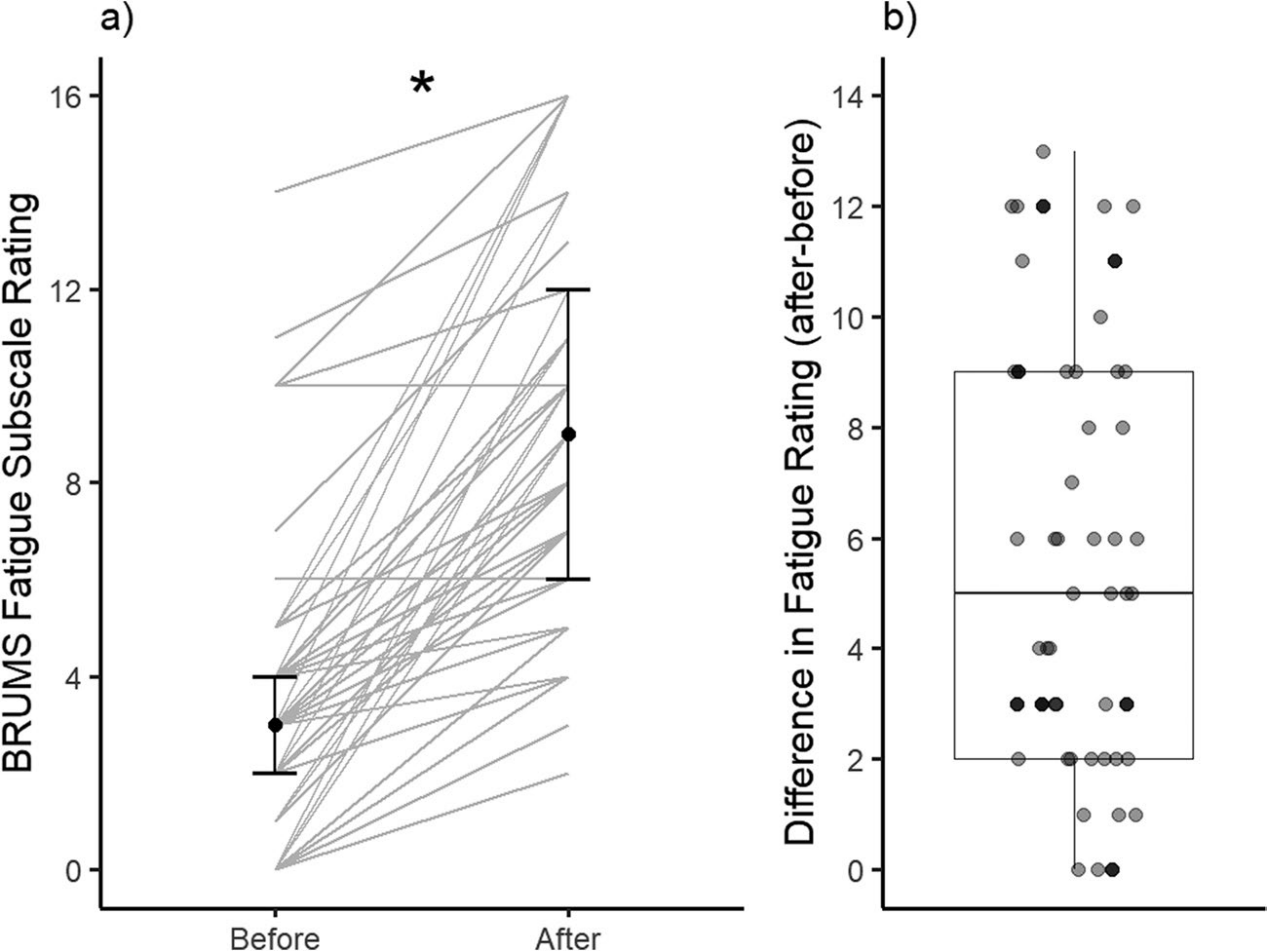
BRUMS Fatigue Subscale Rating and Difference in BRUMS Fatigue Subscale Rating before and after the mental fatigue battery (*n* = 45). In **a**, individual responses are represented by *thin gray lines*, with the* black dots *representing the median. The upper and lower horizontal lines indicate the first and third quartiles (25th and 75th percentiles). In **b**, individual responses are plotted as a *single dot*. The values in **b** are calculated as post-pre, meaning that values above zero indicate participants who reported higher feelings of fatigue at the end of the mental fatigue battery than they did at the beginning. The upper and lower whiskers (*vertical lines*) of the box plot extend to the range of the data, with there being no outliers. The *top and bottom horizontal lines* indicate the first and third quartiles (25th and 75th percentiles), with the *middle line* showing the median. * denotes a significant difference

A Wilcoxon signed-rank test with time point (pre/post) as the independent variable and BRUMS fatigue subscale score as the dependent variable revealed a significant increase in subjective feelings of fatigue, *z* = – 5.72, *p* < 0.001, 95% CI [4.5, 7], *r* = .85. Participants reported feeling significantly more fatigued after completing the cognitive task battery than they were before they started the battery (Fig. 6). This allowed us to accept our hypothesis (a) that completing two hours of a cognitive task battery will cause an increase in subjective feelings of fatigue. Out of 45 participants, 43 reported an increase in subjective fatigue, with two participants reporting no change.

### AX-CPT performance

**Fig. 7:**
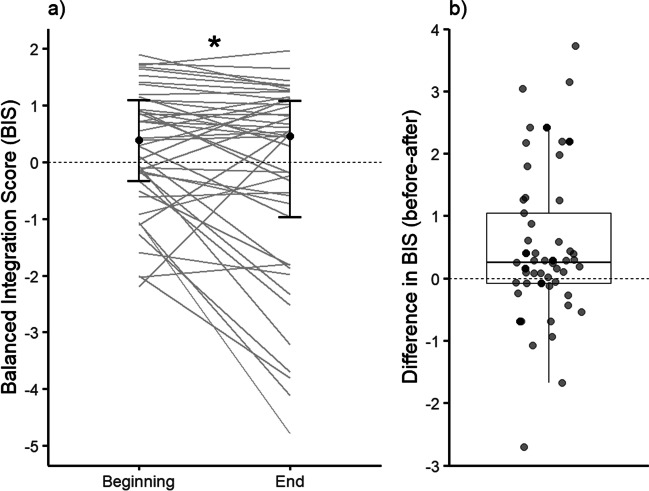
BIS Score and Difference in BIS Score in the AX-CPT Task at the beginning and end of the mental fatigue battery (*n* = 45). In **a**, individual responses are represented by *thin gray lines*, with the *black dots* representing the median. The *upper and lower horizontal lines* indicate the first and third quartiles (25th and 75th percentiles). In **b**, individual responses are plotted as a *single dot*. The values in **b** are calculated as pre-post, meaning that values above the zero line are participants who performed worse at the end of the mental fatigue battery. The upper and lower whiskers (*vertical lines*) of the box plot extend to 1.5 times the interquartile range, with points outside these whiskers being outliers that were not removed from the data. *Top and bottom horizontal lines* indicate the first and third quartiles (25th and 75th percentiles), with the *middle line* showing the median. * denotes a significant difference. For a figure summarizing the BIS across all repeats of each cognitive task, please see Appendix Fig. 9

A Wilcoxon signed-rank test with time point (pre/post) as the independent variable and AX-CPT BIS as the dependent variable revealed a significant decrement in task performance, *z* = – 2.64, *p* = 0.008, 95% CI [.09, .75], *r* = .39. Participants’ performance in the AX-CPT was significantly worse at the end of the cognitive task battery in comparison to their performance at the beginning (Fig. 7). This allowed us to accept our hypothesis (b) that completing two hours of a cognitive task battery will cause a decrement in cognitive task performance.

## Exploratory analyses

### Other BRUMS subscales

**Fig. 8:**
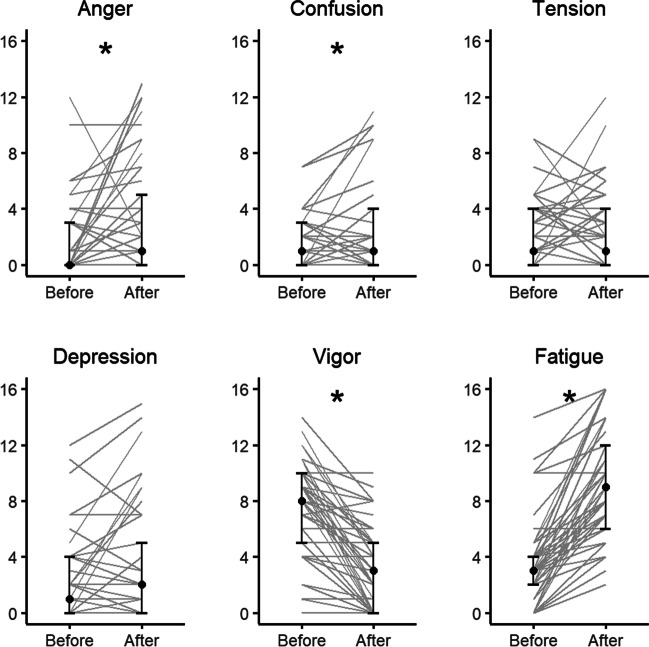
Participants’ responses in all BRUMS subscales before and after the mental fatigue battery: anger, confusion, tension, depression, vigor, fatigue (*n* = 45). Individual responses are represented by thin *gray lines*, with the *black dots* representing the median. The *upper and lower horizontal lines* indicate the first and third quartiles (25th and 75th percentiles). * denotes subscales with significant differences

A decrease in vigor has previously been identified as an important marker of mental fatigue (Pageaux et al., 2015), so we chose to test the apparent decrease statistically. A Wilcoxon signed-rank test revealed a significant decrease in subjective feelings of vigor, *z* = – 5.39, *p* < 0.001, 95% CI [– 5.5, – 3.5], *r* = .80 (Fig. 8). A Spearman’s rank correlation exploring the changes in vigor and fatigue also revealed a significant negative correlation, *r* = – .52, *p* < 0.001. Overall, participants experienced decreases in vigor from before to after the cognitive task battery. Participants who experienced greater increases in fatigue also experienced greater decreases in vigor.

Further investigation of the remaining BRUMS subscales showed that participants did not only report changes in fatigue. A series of Wilcoxon signed-rank tests revealed statistically significant increases in anger, *z* = – 3.15, *p* = 0.002, 95% CI [0.99, 4], *r* = .47, and confusion, *z* = – 2.16, *p* = 0.031, 95% CI [0.00005, 2], *r* = .32. There were, however, no changes found in depression, *z* = – 1.73, *p* = 0.084, 95% CI [– 2.6, 3], *r* = .26, or tension, *z* = – 0.19, *p* = 0.851, 95% CI [– 1.5, 1], *r* = .03.

## Discussion

The aim of this paper was to develop and validate a novel method to induce mental fatigue. The task we developed was successful at inducing both an increase in subjective feelings of fatigue and a decrement in cognitive task performance, supporting both our hypotheses. Our task therefore seems suitable for inducing mental fatigue as defined by Marcora et al. (2009) – participants experienced subjective feelings of fatigue (including but not limited to tiredness and a lack of energy), and their cognitive task performance decreased over time indicating that task demands were high.

The subjective increase in mental fatigue that we observed using the BRUMS fatigue subscale is slightly larger than that reported by Marcora et al. (2009), who found that when participants completed a continuous 90-min AX-CPT task, they experienced an increase from a mean of approximately 4 ± 4 to 7 ± 5, in comparison with 4 ± 3 to 9 ± 4 in this study. This indicates that our novel task can induce greater fatigue in participants than previous approaches, possibly due to the longer task duration. Given the heterogeneity of approaches in the current literature, in particular with different approaches to measuring subjective fatigue (e.g., by using different types of visual analogue scale (O’Keeffe et al., 2020; Smith et al., 2016), and the fact that there is no standardized way of reporting subjective fatigue outcomes, it is difficult to directly compare the size of our effect with that found in the literature more broadly. The decrement in cognitive task performance that we observed was significant, but the effect was relatively small. Again, it is difficult to compare this result to that found in the broader literature, as we used an integrated measure of reaction time and accuracy (the BIS), where previous literature has not. For example, we could compare changes in task accuracy in this study with changes in the accuracy in Marcora et al. (2009) – reductions of 3.5% and 5.9% – but this is not meaningful due to different population types and sizes, the different experimental approaches, and the possibility of unaccounted changes in reaction time. Future researchers should use integrated measures such as the BIS to examine cognitive task performance to account for speed–accuracy trade-offs.

Some participants’ performance in the AX-CPT appeared to improve as a result of our manipulation, with 14 out of our 45 participants performing better in the AX-CPT in the final 10 min of the cognitive battery than in the initial 10 min. Whilst all these participants nonetheless experienced an increase in subjective fatigue, this highlights the need for researchers to carefully consider other factors when assessing mental fatigue such as behavioural task performance (Van Cutsem et al., 2017). There are many possible reasons why these participants’ performance might have improved. For example, as this study was conducted remotely, it is possible that participants were not fully engaged at the beginning of the task. Also, nine participants completed the testing session more than 2 days after the training session, and consequently may have needed time to remember how to perform the task, resulting in worse initial performance. This could have led to an apparent improvement in performance over time instead of a decrease in performance. However, if this effect was present in our data, it did not impact the overall outcome which was to find a decrement in task performance from the beginning to the end of the task battery. Similarly, a learning effect may have taken place, where participants improved due to practice over the 2-h time period of the battery, or this sub-group of participants may have independently monitored their elapsed time very closely allowing them to know the end-point of the task and invest more effort in the final 10 min (Katzir et al., 2020), where other participants did not monitor their time closely enough to provide them with this information. Under the definition of fatigue used in this study, participants who experienced a subjective increase in feelings of fatigue with no concomitant performance decrement would still qualify as being mentally fatigued, as the level of cognitive demand placed on participants could be assumed. This interpretation, however, should be used with caution in a scenario where an experimenter is interested in examining the effects, correlates, or markers of mental fatigue, as subjective reports are subject to experimenter demands (Thompson et al., 2019).

In accordance with prior research into mental fatigue (Boksem & Tops, 2008), we found a great variety of emotional responses were reported across the BRUMS subscales for anger, confusion, tension, and depression. This supports the notion of mental fatigue as being related to mood change (O’Keeffe et al., 2020). From these exploratory findings, we suggest that mental fatigue is a multifaceted experience which is not limited simply to changes in fatigue and vigor or changes in task performance. Participants’ differing mood states could differentially affect their performance in subsequent or concurrent tasks, give misleading neural correlates of mental fatigue, or cause them to conflate fatigue with other feelings. It is important that the way fatigue is measured and operationalized is sensitive to these possible confounding variables.

Given our experiences with participant withdrawals and exclusions, and the wide range of scores in the AX-CPT, we recommend that experimenters aiming to induce fatigue in participants should supervise participants (as opposed to their completing the task remotely), motivate them to perform well (e.g., by providing a monetary reward), and exclude participants who do not appear to engage with the task (as we have done, or for example by using attention checks). Disengagement may be an indicator of mental fatigue especially when the disengagement is towards the end of fatigue-inducing task. In the current study, however, it is difficult to know the reason for disengagement as participants completed the study remotely and without supervision.

## Limitations

In this work, we did not conduct a replication of our findings. Researchers who are interested in using this task in their own work may wish to reproduce this study in order to better understand the replicability of the effects. This is especially relevant for researchers who might use different populations or for researchers using smaller sample sizes, as we identified varied behavioural responses across our broad range of participants. Researchers who are interested in the transfer of behavioural effects of mental fatigue may also wish to expand future replications to examine the effects of this task battery on a wider range of cognitive tasks (i.e., not only in the AX-CPT).

The current task design does not account for participants’ perceptions of boredom of or mental effort. According to theories of underload (Van Cutsem et al., 2022), boredom should not be a risk in the current study as the cognitive challenge was high. Future research should seek to evaluate participants’ levels of boredom as related to the implementation of the task battery, in order to identify and possibly adjust for any confounding affects. Related to boredom, mental effort is also an important aspect of mentally fatiguing tasks which was not directly measured in this study. Future research should measure mental effort rather than using assumed or inferred methods. A simple test such as the NASA Task Load Index (National Aeronautics and Space Administration, 2022) could measure mental effort as well as other potentially relevant outcomes such as perceptions of physical effort and task performance, which are not captured in the BRUMS. This would allow researchers to account for additional confounds that could be present if, for example, participants perceive physical fatigue as a consequence of the mental fatigue manipulation.

Finally, the online nature of our study meant that we were unable to practically control for extraneous factors such as participants’ sleep quality, caffeine or alcohol consumption, or physical illness. It is possible that this has added noise to our data, which future researchers may wish to avoid, especially if they are using smaller sample sizes.

## Conclusion

In conclusion, we have shown that this novel task battery is capable of inducing subjective increases in feelings of mental fatigue and a concurrent decrement in cognitive task performance. This method addresses issues of heterogeneity and lack of ecological validity in the current literature. By moving towards a unified way of inducing fatigue, the scientific literature on mental fatigue would move towards a greater consensus, allowing for comparison between studies and easier collaboration across different disciplines. This novel method provides an approach which could be used to test the effects, theoretical nature, and physiological markers of mental fatigue.

## Figures and Tables

**Fig. 9:**
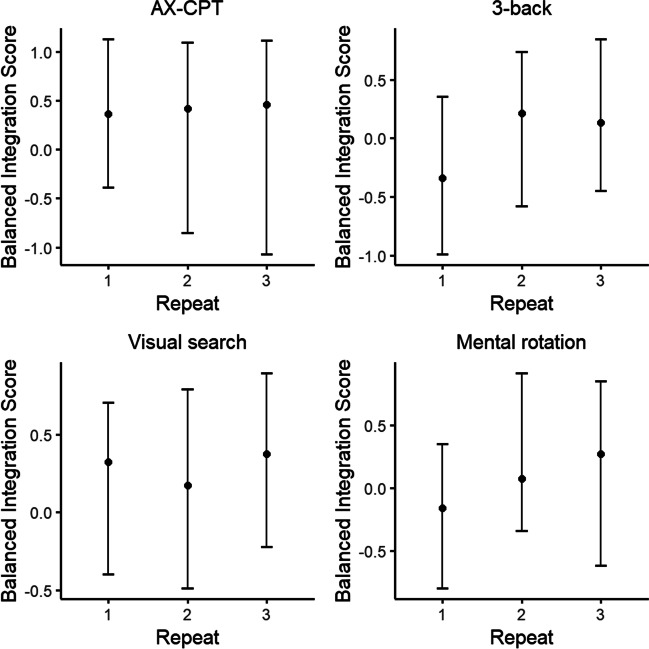
BIS outcomes in all four tasks: AX-CPT, 3-back, visual search, and mental rotation (*n* = 45). *Black dots* represent the median score with the *upper and lower horizontal lines* indicating the first and third quartiles (25th and 75th percentiles)

## Data Availability

The data and materials for the experiment reported here are available at https://osf.io/6hjc3/ and the experiment was preregistered.

## Abbreviations

AX-CPT: A-X Continuous Performance Test
BIS: Balanced Integration Score
BRUMS: Brunel Mood Scale

## Code availability

The datasets generated and analysed during the current study, as well as the study materials, analysis code, figures, and pre-registration document, are available in the associated Open Science Framework repository at https://osf.io/6hjc3/
